# Lipid-Based Nanoparticulate Systems for the Ocular Delivery of Bioactives with Anti-Inflammatory Properties

**DOI:** 10.3390/ijms232012102

**Published:** 2022-10-11

**Authors:** Raquel da Ana, Joel Fonseca, Jacek Karczewski, Amélia M. Silva, Aleksandra Zielińska, Eliana B. Souto

**Affiliations:** 1 Department of Pharmaceutical Technology, Faculty of Pharmacy, University of Porto, Rua Jorge de Viterbo Ferreira, 228, 4050-313 Porto, Portugal; 2 Department of Environmental Medicine, Poznan University of Medical Sciences, Fredry 10, 61-701 Poznan, Poland; 3 Department of Gastroenterology, Dietetics and Internal Diseases, Poznan University of Medical Sciences, Fredry 10, 61-701 Poznan, Poland; 4 Department of Biology and Environment, University of Trás-os-Montes e Alto Douro, UTAD, Quinta de Prados, 5001-801 Vila Real, Portugal; 5 Centre for Research and Technology of Agro-Environmental and Biological Sciences, CITAB, UTAD, Quinta de Prados, 5001-801 Vila Real, Portugal; 6 Institute of Human Genetics, Polish Academy of Sciences, Strzeszyńska 32, 60-479 Poznan, Poland; 7 REQUIMTE/UCIBIO, Faculty of Pharmacy, University of Porto, Rua Jorge de Viterbo Ferreira, 228, 4050-313 Porto, Portugal

**Keywords:** ocular drug administration, lipid nanoparticles, anti-inflammatory drugs, immunosuppressive drugs, antioxidants

## Abstract

The complexity of the eye structure and its physiology turned ocular drug administration into one of the most challenging topics in the pharmaceutical field. Ocular inflammation is one of the most common ophthalmic disorders. Topical administration of anti-inflammatory drugs is also commonly used as a side treatment in tissue repair and regeneration. The difficulty in overcoming the eye barriers, which are both physical and chemical, reduces drug bioavailability, and the frequency of administration must be increased to reach the therapeutic effect. However, this can cause serious side effects. Lipid nanoparticles seem to be a great alternative to ocular drug delivery as they are composed from natural excipients and can encapsulate both hydrophilic and lipophilic drugs of different sources, and their unique properties, as their excellent biocompatibility, safety and adhesion allow to increase the bioavailability, compliance and achieve a sustained drug release. They are also very stable, easy to produce and scale up, and can be lyophilized or sterilized with no significant alterations to the release profile and stability. Because of this, lipid nanoparticles show a great potential to be an essential part of the new therapeutic technologies in ophthalmology to deliver synthetic and natural anti-inflammatory drugs. In fact, there is an increasing interest in natural bioactives with anti-inflammatory activities, and the use of nanoparticles for their site-specific delivery. It is therefore expected that, in the near future, many more studies will promote the development of new nanomedicines resulting in clinical studies of new drugs formulations.

## 1. Introduction

Ocular drug delivery remains one of the most challenging routes of drug administration in the pharmaceutical field mainly because of the complex nature and structure of the eye. Barriers such as epithelial, aqueous–vitreous, blood-aqueous barrier (BAB), and blood-retinal barriers (BRB) can limit drug delivery through different routes to the eye. Most drugs are formulated as eye drops and ointments for ophthalmic use. They are cost-effective, patient compatible, and simple in the formulation. However, once the drug is applied topically, it is easily washed with tears or removed by other mechanisms, leading to the need for several applications in a day to achieve a therapeutic effect [1]. It can induce unwanted systemic effects [2]. Nanotechnology is a promising field that allows the improvement of the therapeutic efficiency, compliance, and safety of ocular drugs. Lipid-based nanocarriers are one of the most interesting colloidal drug delivery systems once they are biodegradable and biocompatible. Therefore, they have secured the title of nanoscale carriers [3]. Ocular inflammation is one of the most common ocular diseases, resulting from various causes. Eye inflammation occurs in response to infection, allergies, autoimmune disorders, or injury trauma. Treating ocular inflammation includes pharmacotherapy with corticosteroids, non-steroidal anti-inflammatory drugs (NSAIDs) for anterior scleritis, and immunosuppressive drugs. More recently, biological agents, such as inhibitors of tumor necrosis factor alpha (TNF-α), have been tested [4]. solid lipid nanoparticles (SLNs) and nanostructured lipid carriers (NLCs) are interesting carriers for ophthalmic applications [2]. SLNs and NLCs can be designed to treat the most critical ocular disorders, such as joint ocular drug inflammation or infection, glaucoma, or diseases that affect posterior eye structures. These systems are an innovative approach that has been considered a promising strategy for treating some disorders in the retina [4]. They are interesting for ocular drug delivery because they improve corneal permeation and increase bioavailability. Besides these, lipid nanoparticles (LNs) are also safe, non-invasive, enhance therapeutic benefits because of the increased residence time at the administration site, and have small or no local side effects [5]. Other attractive characteristics are the therapy efficiency, compliance, safety of ocular drugs, and compatibility and versatility [2]. Furthermore, one of the essential properties of lipid nanoparticles to apply in ocular drug delivery is the adhesive properties, mainly due to their small size. The adhesive properties depend highly on the surface properties, especially mucosal surfaces. Surface modifications of the particles are used as a strategy to prolong the contact time with the cornea. These surface modifications can be achieved using phospholipids, chitosan, cysteine-polyethylene glycol stearate conjugate, and stearylamine. Cationic lipids, polysaccharide emulsifiers, or other moieties with cationic groups have been included in the lipid nanoparticle composition to enhance mucoadhesion with anionic ocular tissues by electrostatic adhesion [4]. The electrostatic attraction between the cationic nanoparticles and the anionic cellular components of the ocular surface tissues enhances the bioavailability of the drugs delivered by means of LNs. In this way, considering their intrinsic capacity to adhere to the ocular surface and the interaction with the epithelium, LNs could be an essential part of new therapeutic technologies in ophthalmology [2,6]. 

Moreover, considering the general requirement for ophthalmic formulations regarding their aseptic conditions and sterilizations, LNs have the advantage that they can be lyophilized and sterilized by heat with only minor effects on their stability and in vivo performance. Additionally, they can be produced on a large scale and present long-term stability [2].

Over the last few years, the use of LNs, e.g., SLN/NLC, liposomes, nanoemulsions, has gained great interest for the ocular delivery of synthetic, biological or natural compounds with anti-inflammatory activity. Natural compounds are considered as an alternative over synthetic drugs, attributed to their range of physicochemical properties, making them interesting options for the treatment of ocular disorders. 

## 2. Overcoming Obstacles in Ocular Drug Delivery—Ocular Anatomy and Challenges in Drug Delivery

**Figure 1:**
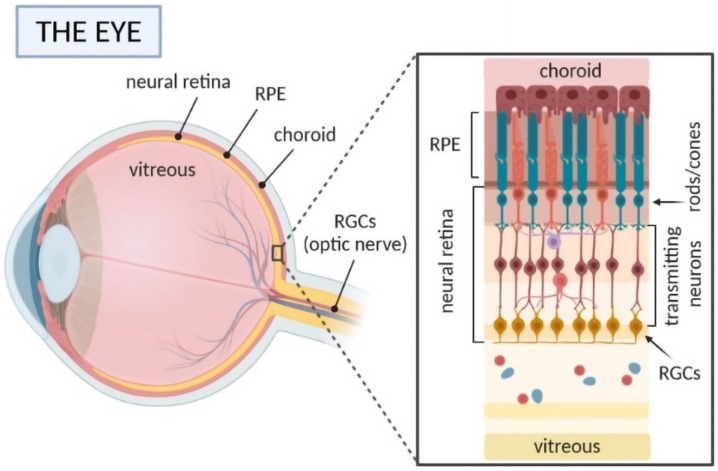
Anatomy of the human eye (own drawing). Abbreviations: RGCs—retinal ganglion cells; RPE—retinal pigment epithelial.

The challenges of ocular drug delivery are mainly related to the complex structure and nature of the eye, as shown in Figure 1 [1]. The specific physiological and anatomical features make it a complicated and sophisticated organ, as several barriers must be overcome to reach a particular ocular tissue [7]. The human eye, a globular organ, consists of two major parts: the anterior and posterior segments. Considering their many biological barriers for both parts, they can be generally divided into categories: anatomical barriers are divided into static and dynamic barriers. Static barriers are related to the corneal epithelium and blood-aqueous barrier, and dynamic barriers include tear drainage and conjunctival blood and lymph flow. On the other side, physiological barriers include metabolic and intraocular environment barriers, where the blood-aqueous barrier (BAB) and blood-retina barrier (BRB) are found [8,9]. BRB is a crucial structure that maintains homeostasis in the posterior segment of the eye. It is composed of the retinal pigmented epithelium in the outer part, and in the inner part the endothelium of capillaries in the retina is found [10]. BAB and BRB pose significant limitations for drug delivery via different routes to the eye [1]. 

Considering the target segment, several administration routes are available (Table 1). Topical administration is the preferred route for treating diseases affecting the anterior part of the eye. Indeed, conventional approaches in ocular delivery include solutions, suspensions, and ointments, comprising almost 90% of the available ophthalmic formulations on the market. They offer significant advantages, such as ease of formulation, drug delivery, and low preparation costs. They are also easy to use, which leads to high patient compliance and cost-effectiveness [11,12,13]. When the aim is the delivery of active compounds to other tissues like the retina, different administration routes are needed, such as systemic, intraocular, periocular, or intravitreal administration [6]. However, when considering topical administration, the therapeutic efficacy of drugs formulated as conventional eye drops is minimal because of the anatomical barriers and physiological conditions that protect the eye against the penetration from foreign substances [14]. Firstly, a significant fraction of the drug applied topically is washed away with tears or removed by other mechanisms. It requires frequent administration of the ophthalmic preparation to achieve therapeutic efficacy for treating diseases in the eye’s posterior segment. This leads to limited drug residence time over the cornea, which reduces drug absorption and increases cytotoxicity [1,10]. Some other limitations of eye drops are low drug bioavailability, the impossibility of targeting specific ocular structures, and drug binding or inactivation by tear proteins. Moreover, most active compounds show unfavorable physicochemical properties that prevent absorption and distribution throughout the ocular tissues, dramatically decreasing the fraction of drug that reaches the target tissue [14].

Literature reports that the ideal formulation for topical ocular drug delivery may be able to increase the drug residence time at the ocular surface, protect the drug from degradation by tear enzymes, provide targeted drug delivery to the active site, increasing the bioavailability and minimizing the non-productive absorption, and finally promote drug penetration through the cornea to enable it to act as a controlled release system and consequently reducing the dosing frequency. The materials used in the ocular drug delivery system must be biocompatible, biodegradable, and non-irritating [14]. In this way, it is possible to see that physicochemical properties such as particle size, surface net charge, shape, solubility, degree of ionization, and lipophilicity influence the ocular drug absorption and determine the administration route. These referred factors can be tailored by using novel particulate drug delivery systems that enhance the bioavailability of ocular drugs [1]. 

## 3. Application of Nanotechnology in Ocular Drug Delivery

Nanotechnology is changing the perception of drug administration using conventional dosages. It can revolutionize the way new therapies are developed and optimize the existing ones by combining science and technology and the ability to manipulate structures and properties at the nanoscale range [1,9]. Nanocarriers have been shown effective in overcoming the limitations of current therapies [12]. The term nanoparticles refers to a particulate drug delivery system with particle size in the nanometre range (1–1000 nm) [12]. Nanoparticle technologies, in general, show several benefits as solubilization of hydrophilic and poorly water-soluble drugs, improvement of bioavailability and pharmacokinetic properties, and protection of the drug from physical, chemical, and biological degradation. The sub-micrometer size of these systems also allows efficient transportation and crossing of natural eye barriers, which leads to appropriate drug delivery to the target site [2,9]. Some chronic inflammatory diseases, such as age-related macular denegation (AMD) and uveitis, require drug maintenance at specific concentrations, which is an essential point in the treatment [15]. Particle size, particle size distribution, and stability are some of the most significant issues in formulating dispersed systems for ocular administration. Other potential advantages of nanoscale drug delivery systems in ocular therapy are the possibility of self-administration by patients as eye drops, no impairment of sight because of small dimensions of the delivery systems, possible uptake into corneal cells, and targeting toward affected tissues, reducing potential side effects and required doses [16]. All the reported advantages and limitations of lipid-based nanoparticulate systems can be found in Table 2. 

**Figure 2:**
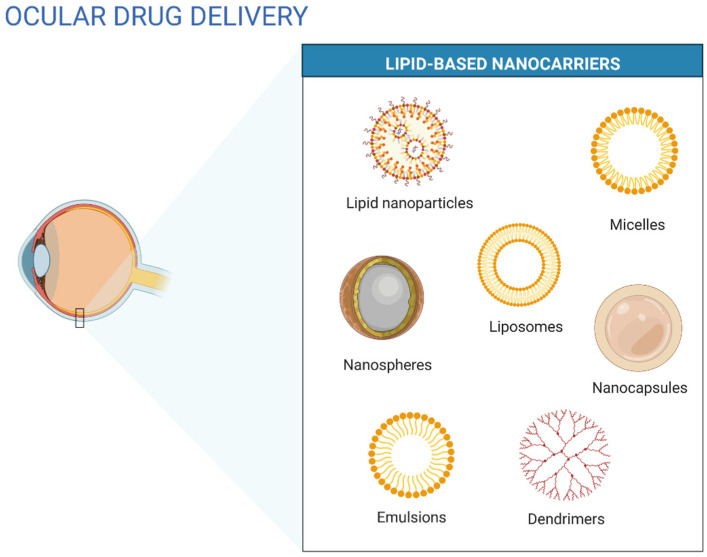
Lipid-based nanostructured carriers for ocular drug delivery (own drawing).

Colloidal drug delivery systems can be easily administered in liquid form. Colloidal systems include suspensions of microparticles, liposomes, or nanoparticles [17]. Other nanostructured systems such as SLNs, niosomes, nanocapsules, nanospheres, dendrimers, nanosuspensions, and nanoemulsions have also been used in ocular drug delivery (Figure 2) [1]. 

Liposomes are small artificial vesicles produced by natural phospholipids and cholesterol [6]. Some of the advantages of liposomes are their low toxicity and antigenicity, the capacity to be biodegraded and metabolized in vivo, and their liposomal properties which can be controlled to some extent, such as membrane permeability. Liposomes can also entrap and protect drugs on the journey to the target site [18]. However, they have some associated problems, starting with their liquid form, which limits their pharmaceutical formulation feasibility. Most methods of sterilization are also unsuitable for liposomes. Heating involved in autoclaving can irreversibly damage their vesicular structure, and filtration reduces the particles to an average of 200 nm, limiting their application [1]. LNs have a higher loading capacity and show higher biological and storage stability than liposomes [6]. LNs are colloidal structures with submicrometer sizes, usually between 50 and 400 nm. They are made of biocompatible and biodegradable lipids, such as glycerides, fatty acids, waxes, and derivatives, that are stabilized by surfactants and co-surfactants when required [14]. Lipid components of lipid-based drug delivery systems show similar properties to those of the tear film. They can interact with the outside lipid layer of the tear film, allowing the increase of residence time of the carrier in the conjunctival sac, which acts as a drug depot [19].

The most suitable drugs for lipid formulations are the ones with significant lipophilicity, which means a logP of at least 2. Additionally, the optimal drug physicochemical properties are neutral or base drugs, with low melting temperature (<150 °C), some polar functional groups, and adequate solubility of the drug in lipids and water [10,20]. Considering the different types of LNs, the first generation is related to SLNs. They result from the technological evolution of oil-in-water nanoemulsion, replacing the liquid lipid of the emulsion droplets with solid lipids at room temperature. These nanocarriers have more physical stability than nanoemulsions once the solid structure is formed by a rigid core surrounded by a stabilizing surfactant layer. Surfactants are used at a lower concentration than in nanoemulsion preparations. This results in less toxicity and a better biocompatibility profile. However, because of their rigid crystalline matrix, SLNs show some drawbacks. The two main limitations are the low drug loading and drug leakage during storage. To overcome these technological limitations, the second generation of lipid nanoparticles has been developed [14]. NLCs are lipid nanoparticles characterized by a solid lipid core consisting of a mixture of solid and liquid lipids. The resulting matrix of lipid particles exhibit a lower melting point compared with SLNs, but the matrix remains solid at body temperature [3]. The goal of the addition of a liquid lipid is to increase the molecular disorganization of the lipid lattice and consequently increase the payload and prevent drug expulsion during storage. Even though the resulting structure usually contains up to 30% of liquid lipids, the solid state is maintained without crystalline formation in the lipid matrix. When compared with other lipid-based nanocarriers, lipid nanoparticles offer several pharmaceutical advantages. They exhibit excellent biocompatibility and biodegradability because they use generally recognized as safe (GRAS) substances. They have high physical stability during storage and biological stability in environments with intense enzymatic activity. LNs are also easy to modulate their physicochemical characteristics. They can be produced by solvent-free methods and undergo sterilization by autoclaving. Because of all these characteristics, LNs are very attractive to industry [14].

## 4. Ocular Inflammation

**Figure 3:**
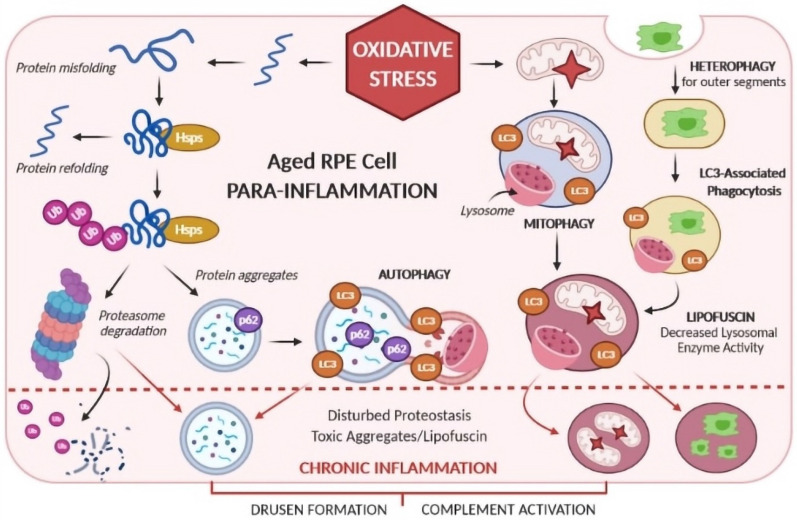
Age retinal pigment epithelium (RPE) cell with inflammation (own drawing). Abbreviations: Hsps—Heat-shock proteins; LC3—long-chain protein 3; Ub—ubiquitin; p62—an autophagy marker.

One of the most common disorders in ophthalmic therapy is the ocular inflammatory disease that affects any part of the eye or the surrounding tissues. Eye infection is a common problem, observed in all ages, that can be caused by different microorganisms, such as bacteria, fungi, or viruses. Inflammation involving the eye can range from the familiar allergic conjunctivitis of hay fever to rare, potentially blinding conditions such as keratitis, scleritis or episcleritis, uveitis, optic neuritis, and others [2]. Ocular inflammation can be the result of a wide variety of causes, including infections and inflammatory disorders. Ocular joint inflammation involves the sclera (episcleritis) and the uvea (uveitis) [4]. Most ocular diseases and surgeries are related to an inflammatory response [15]. Depending on the area of the uvea affected, namely the anterior (iritis, iridocyclitis), the intermediate (pars planitis), the posterior (choroiditis, chorioretinitis), or the global structure (panuveitis), ocular damage can be induced. Despite the eye-specific target site and the specialized ocular medicine to direct eye treatment, most cases of local inflammation that reach the inner eye structures are treated by intraocular injections [15]. Regarding the drug treatment approach, ocular inflammation includes corticosteroids, NSAIDs, and immunosuppressive drugs. Recently, biologic agents such as TNF-α have also been tested [4]. Although steroidal agents have been the standard treatment for ocular inflammation, the use of NSAIDs has increased over the past years. Some clinical evidence shows a synergistic effect of the combined use of NSAIDs and steroids. The use of topical NSAIDs allows the undesirable impacts of steroidal agents, namely, the decrease of immunological response to infection, cataract formation, steroid-induced raised intraocular pressure, and inhibition of re-epithelization followed by epithelial denudation [2]. Figure 3 describes the mechanisms of age retinal pigment epithelium (RPE) cell with inflammation.

### 4.1. Corticosteroids

Corticosteroids suppress the inflammatory response against various inciting agents of mechanical, chemical, or immunological nature. They are usually considered more substantial than other options and superior at dealing with inflammation, primarily associated with cataract surgery. Steroids can also interact with specific DNA sequences of the cellular nucleus, which alter the production and inhibitory proteins and inhibits additional inflammatory mediator production. 

Topical corticosteroid solution eye drops are generally ineffective for treating posterior segment inflammation. Topical drug exposure at the therapeutic levels happens only for a short time-frame; thus topical steroid therapy must be carried out for several weeks after surgery until the blood-aqueous barrier is re-established. Other alternatives have been found, such as subconjunctival injection that can achieve higher intraocular steroid levels or systemic therapy. Intravitreous injection of corticosteroids is another approach for intraocular inflammation. 

Steroids have the most potent efficacy in inflammation treatment. However, they can induce significant side effects. Short-term use helps minimize the risk while reaping substantial benefits. However, some of the most problematic side effects caused by the long-term use of topical steroids include ocular hypertension, glaucoma, and cataract formation. Other side effects of steroids include mydriasis, ptosis, inhibition of corneal epithelial or stromal healing, punctate staining, damage to the optic nerve, and defects in visual acuity and visual fields [2].

Dexamethasone is one of the most commonly used drugs for the treatment of eye diseases. It is a high-efficacy glucocorticosteroid and can be applied in inflammatory eye diseases for both anterior and posterior segments. Despite it being one of the most potent anti-inflammatory drugs, it can have severe side effects on non-target organs [21,22,23].

### 4.2. Non-Steroidal Anti-Inflammatory Drugs (NSAIDs)

NSAIDs are drugs with analgesic properties used to treat acute or chronic conditions involving pain and inflammation. These analgesic properties are linked to the inhibitory activity of cyclooxygenases (COX) [24]. Due to the ability to avoid the undesirable side effects of steroidal drugs, the use of NSAIDs has increased in the past years, even though steroidal agents are considered the classical treatment of ocular inflammation. NSAIDs are commonly used in topical administration in the management and prevention of ocular inflammation that involves structures of the anterior segment of the eye [4]. Specifically, they are used in managing post-operative inflammation, inhibition of intraoperative meiosis, treatment of seasonal allergic conjunctivitis, and pain control. NSAIDs have also helped decrease bacterial colonization related to contact lenses and prevent bacterial adhesion to human corneal epithelial cells. Recently, they have been used inflammatory surface reactions, like dry eyes, based on the immune response. The topical use of NSAIDs in ophthalmology is limited to relatively water-soluble acids, with most NSAIDs drugs being weakly acidic drugs that ionize in lacrimal pH fluid and have limited permeability through the anionic cornea. Reducing the pH of the formulation leads to the non-ionized fraction of the drug, allowing for enhanced permeation. However, this can potentially increase irritation. Thus, the design of NSAID formulations that are comfortable for topical eye application is required [2]. Drugs, such as dexamethasone and ibuprofen, have already been loaded in nanostructures, and drug efficiency was shown to been improved and maintained at particular concentrations in ocular tissues [15].

### 4.3. Immunosuppressive Agents

Immunosuppressive drugs have been widely used to control severe ocular infections [25]. These drugs have been recommended for treating inflammatory disorders resistant to local and oral corticosteroid therapy or to avoid the problematic side effects induced by the long-term treatment with these drugs [4]. Immunosuppressive therapy for the treatment of ocular infections is thus only recommended when corticosteroid therapy—the first line treatment—fails to control inflammation, when topical corticosteroid-sparing therapy is needed to minimize the side effects of systemic corticosteroids, and for specific diseases that show a better response to the early initial use of non-corticosteroid immunosuppression [26]. It is possible to categorize four main immunosuppressive agent classes: antimetabolites, T cell inhibitors, alkylating agents, and biological response modifiers [26]. Antimetabolites include drugs such as methotrexate and mycophenolate mofetil. Cyclosporine and Tacrolimus are examples of T cell inhibitors, while for alkylating agents the main examples are cyclophosphamide and chlorambucil. The initial choice of drug depends on the patient’s comorbidities and age [27]. Some ocular inflammatory diseases, such as severe scleritis and uveitis, require immunomodulatory therapies, otherwise they could result in severe damage. For complicated ocular inflammation, immunosuppressive agents, such as cyclosporine A, can be used as an alternative or combined therapy [4]. Azathioprine is another immunosuppressive drug used for treating corneal graft rejections and non-infectious ocular conditions. It can be combined with other immunosuppressive agents in specific cases [25]. These therapeutic agents with biological properties include monoclonal antibodies and soluble cytokine receptors that are regarded as natural response modifiers. The leading biologics currently used are TNF-α, infliximab, adalimumab and etanercept, cytokine receptor antibodies (Daclizumab), and interferon-α (IFN-α) [27].

## 5. Lipid-Based Nanosystems Applied in Ocular Delivery

Lipid-based nanosystems comprise all the lipid nanostructures for drug delivery, from liposomes to micelles, lipid nanoparticles and nanoemulsions, as mentioned above.

**Figure 4:**
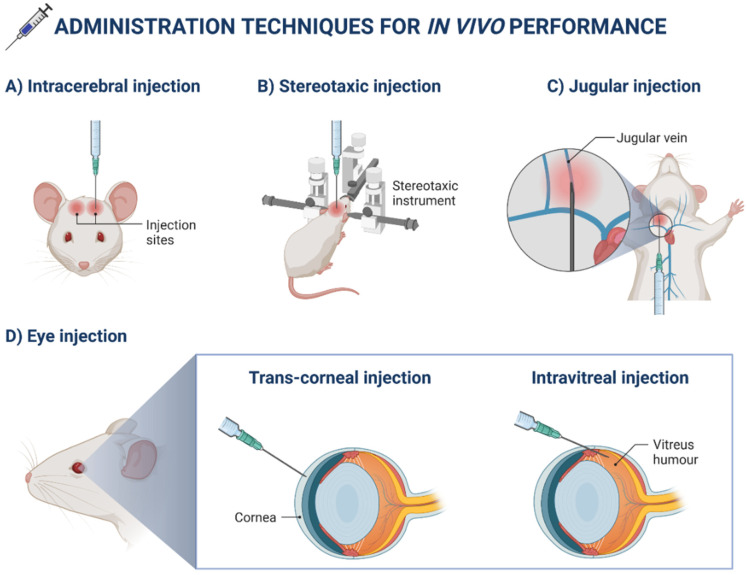
Drug administration techniques for in vivo performance in mice.

Although nanosuspensions/nanoemulsions and polymeric nanoparticles have been successfully developed for several steroidal and NSAID delivery, there are a few drugs that have been formulated in lipid-based nanosystemsto respond to inflammatory ocular disorders. Nowadays, some anti-inflammatory drugs with different chemical structures are being tested. Some examples related to the encapsulation of NSAIDs include the development of SLNs loaded with diclofenac sodium, NLCs containing ibuprofen or flurbiprofen, and finally, for severe ocular inflammatory diseases, cyclosporine A-loaded SLNs [2]. Other research using different lipid-based nanosystems for the ocular administration of drugs to treat ophthalmic inflammation can be found in Table 3. The choice of simpler nanosystems for drug delivery, such as liposomes, nanoemulsions, and micelles, is clear. Additionally, published work has shown that using a polymer as the vehicle is a frequent option to improve the formulation’s physicochemical characteristics and to enhance ocular drug delivery [28]. Due to the mucoadhesive properties and the positive charge, chitosan is one of the most commonly used polymers [29,30]. Nanotechnology-based ocular drug delivery systems, including nanocapsules, microemulsions, liposomes, nanomicelles, SLNs and NLCs, can thus significantly improve the anti-inflammatory drug properties by minimizing the frequency of drug administration, leading to improved compliance. Novel nanoformulations of drugs may be a promising and effective approach for ocular drug delivery in eye infections [31]. Figure 4 exemplifies drug administration techniques commonly used in mice.

## 6. Natural Compounds with Anti-Inflammatory Action

As mentioned above and demonstrated in diverse literature, several anti-inflammatory drugs have been approved for ocular administration, showing tremendous anti-inflammatory responses. However, natural compounds seem to be an excellent alternative for treating several inflammatory ocular disorders. These natural compounds are highly effective because of their many different properties, from anti-inflammatory to antioxidant effects, acting in a complementary way. 

Resveratrol is one of the natural agents mentioned in the literature as a natural compound with anti-inflammatory action used for ocular delivery. It is one of the most well-known phytophagous found in various plants, trees, legumes, and some berries, such as grapes, blackberries, blackcurrants, blueberries, and cranberries. The highest concentration of resveratrol is found in grape skin, making red wine a concentrated source of this natural phenolic compound. The use of resveratrol in ophthalmology is due to its antioxidant, anti-inflammatory, and anti-angiogenic effects. Resveratrol’s anti-inflammatory effects are because of its capacity to limit the expression of pro-inflammatory factors such as interleukins and prostaglandins and decrease the chemoattraction and recruitment of immune cells to the inflammatory site. Moreover, resveratrol also seems to show anti-VEGF (Vascular Endothelial Growth Factor) effects by inhibiting the proliferation and migration of vascular endothelial cells [40]. Work relating to encapsulating resveratrol into lipid-based nanosystems can be found in Table 4. Curcumin is another bioactive compound, yellow-colored, located in the perennial plant *Curcuma longa*. It has a wide range of physiological and pharmaceutical properties: antioxidant, anti-inflammatory, anti-cancer, and neuroprotective. 

Curcumin seems effective in different ocular diseases inhibiting the proliferation of human lens epithelial cells and protecting retinal cells, retinal ganglion cells, and corneal epithelial cells. Due to this, it can be successfully used to treat corneal and choroid neovascularization. However, because of its low solubility, instability, and poor availability the clinical application of curcumin in ophthalmology is still limited. Encapsulating this compound in nanostructures could be an alternative strategy to improve these drawbacks. Li et al. [41] developed a new formulation for curcumin nanomicelles using different polymers [41] (Table 4). Another natural compound is myricetin, a natural flavonol with various biological and pharmacological properties, including anti-inflammatory, antioxidant, and antimicrobial activity. Considering the anti-inflammatory properties, it is particularly beneficial in ocular degenerative and inflammatory diseases, such as dry eye and chronic anterior uveitis. However, some characteristics, such as water insolubility, poor aqueous stability, and poor bioavailability, limit its clinical application. To overcome these drawbacks, Sun et al. [42] designed polymeric micelles to encapsulate myricetin and increase its aqueous solubility, stability, and corneal permeability, promoting its efficacy in the treatment of eye diseases [42] (Table 4). Astaxanthin, a naturally occurring carotenoid, is a bioactive compound with structural and functional characteristics that make it interesting for application in the prevention and treatment of several ocular diseases. This compound can be typically found in marine environments, namely in microalgae and seafood, where it exhibits a red pigment. The most critical biological properties of this compound are the potent antioxidant, anti-inflammatory, and anti-apoptotic activities. Therefore, it has been effectively applied to treat retinal diseases and ocular surface disorders, such as age-related macular degeneration, uveitis, cataract, diabetic retinopathy, and glaucoma [43]. 

## 7. Conclusions

The complexity and physiology of the eye structure, and the drawbacks found in topical ocular administration leads to a need for the development for new technologies for treating inflammatory diseases of the eye. Lipid nanoparticles such as SLNs and NLCs are colloidal systems designed for the ocular administration of anti-inflammatory drugs. Because of their unique properties, they are gaining interest in the pharmaceutical field. Lipid nanoparticles not only improve the therapeutic efficiency, compliance and bioavailability due to their adhesive properties, but they also have high biocompatibility. Furthermore, they are versatile and safe once composed of biocompatible GRAS lipids and produced through solvent-free methods. Lipid nanoparticles can also be used as drug delivery systems to the posterior segment of the eye because of their excellent kinetic stability and controlled drug release properties, thus reducing the frequency of drug administration and, consequently, the possible side effects. Although these is already published research on the use of lipid nanoparticles for the ocular delivery of anti-inflammatory drugs, namely NSAIDs and corticosteroids, it is still quite limited. It is expected that a lot more studies involving the encapsulation of anti-inflammatory drugs in SLNs or NLCs will be published, considering that the marketed formulations is the ultimate goal for ocular drug delivery and their performance in current studies has shown excellent results when drugs are encapsulated in lipid-based nanosystems. Moreover, natural compounds with anti-inflammatory activity seem to be an alternative to standard drugs. They offer a range of biological and chemical properties making them complete and effective in treating ocular diseases. In this way, it is possible to see that in future new formulations for the encapsulation of biocomposites for ocular use will be developed because of their interest and lack of published research in this field. 

## Figures and Tables

**Table 1:** Administration routes for posterior eye delivery.

Route of Administration	Main Routes	Advantages	Disadvantages
Topical	Corneal and conjunctival-scleral pathway	Non-invasive drug delivery and high patient compliance. Minimal systemic side effects.	Low availability due to clearance mechanisms; Short retention time Blurred vision High potential for irritation
Intravitreal	Direct injection into the vitreous humour	Localized drug delivery and maintained at a high therapeutic concentration Minimal off-target effects	Painful process because the injections Frequent injections lead to severe complications
Periocular	Primarily via the trans-scleral pathway	Less painful Bypassing the corneal barrier to achieving adequate therapeutic drug levels The integrity of the eyeball is not affected	Tissue hemorrhage Systemic side effects Rapid clearance
Suprachoroidal	Hollow microneedle injection targeting the choroidal layer	Drug effects at sites maximized by sclera bypassing	High requirements for operation Side effects because of the injections
Systemic	Reach of the choroid through the systemic circulation	Better patient compliance	Low bioavailability Drug-related toxicity because of administration of high doses

**Table 2:** Advantages and disadvantages of lipid-based nanoparticulate systems in ocular delivery.

Advantages	Disadvantages/Limitations
High encapsulation efficiency	Initial burst release from SLNs
High ocular permeation	Low drug loading capacity
Appropriated pharmacokinetic properties	Lack of recent extended clinical trials since most of the studies are just in vivo assessment
Sustained and controlled release	The toxicity of lipid nanoparticles on retinal cells is not entirely studied
Enhancing drug pre-corneal retention time and drug corneal permeability	
Increase ocular bioavailability and distribution	
Prevent ocular toxicity	
Good stability and biocompatibility	

**Table 3:** Summary of lipid-based nanosystems for ocular delivery of anti-inflammatory drugs.

Lipid-Based Nanosystems	Encapsulated Drug	Disease	Administration Route	Main Insights	Ref.
Chitosan-coated liposomes	Triamcinolone Acetonide	Macular Edema	Topical	Improved permeability compared to control suspensions. Prolonged drug residence time on the ocular surface and conjunctival sac by sustained release from the delivery system and reduced pre-corneal drug loss. Good biocompatibility.	[32]
NLC	Triamcinolone Acetonide	Uveitis	Topical	cTA-NLC exhibited slow and sustained in vitro release, good transcorneal permeation ex vivo, and good biocompatibility. The formulation also showed enhanced ocular bioavailability and anti-inflammatory response.	[33]
SLN	Fluticasone	-	Topical	Optimized FP-loaded SLNs displayed an efficient entrapment capacity, small particle size, good stability, and Higuchi release pattern. They also exhibited augmented anti-inflammatory effects when compared with pure and marketed drugs.	[34]
PCL-PEG-PCL micelles	Dexametasone	Uveitis	Injection	DEX-loaded PCL-PEG-PCL micelles showed an improved anti-inflammatory response when compared to the marketed drug once they could reduce the clinical symptoms of uveitis after a lag time. They demonstrate some potential as carriers for DEX in treating anterior uveitis.	[35]
Nanoemulsion	Celecoxib	-	-	NEs increased drug flux through rabbit cornea. They also significantly increased rabbit cornea partitioning, flux, and permeability coefficient. CXB NE formulations can act as permeation enhancers to improve corneal drug delivery.	[36]
Nanoemulsion	Tacrolimus	-	Topical	Optimized CNE formulation exhibited prolonged retention at the corneal surface. In vivo ocular pharmacokinetic studies revealed an increased AUC of the formulation compared to the marketed drug. In vitro cytotoxicity study confirmed the safety of the CNE formulation.	[37]
Chitosan-coated nanoemulsions	Ibuprofen	Dry eye	-	The optimized formulation showed appropriated physicochemical properties for ophthalmic application, good stability, and can be easily sterilized after preparation. The formulation exhibited mucoadhesive properties and excellent biocompatibility. It also provided a prolonged residence time at the ocular surface.	[29]
Liposomes	Flurbiprofen	-	Intravitreous administration	LAP system increased the drug retention time in ocular tissues and high ocular bioavailability. The system showed the capability to decrease inflammatory reactions. It offers good potential for intravitreal drug-sustained delivery.	[38]
Liposomes	Bevacizumab	Choroidal Neovascularization	Intravitreous administration	Bev-MVLs exhibited high encapsulation efficiency and sustained drug release effects in vitro and in vivo. The structural stability of bevacizumab was maintained. The formulation also significantly inhibited the thickness of CNV lesions in in vivo studies.	[39]

Abbreviations: AUC—area under curve; Bev—bevacizumab; CNE—cationic nanoemulsion; CNV—choroidal neovascularization; CXB—celecoxib; DEX—dexametasone; FP—fluticasone propionate; LAP—liposome aggregate platform; MVL—multivesicular liposomes; NE—nanoemulsion; PCL-PEG-PCL—polycaprolactone-polyethylene glycol-polycaprolactone; TA—triamcinolone acetonide.

**Table 4:** Summary of lipid-based nanosystems for ocular delivery of natural compounds with anti-inflammatory action.

Lipid Nanoparticles Type	Natural Compound	Disease	Administration Route	Main Insights	Ref.
PVCL-PVA-PEG nanomicelles	Curcumin	Corneal and choroid neovascularization	Topical	Curcumin PVCL-PVA-PEG nanomicelles had narrow size distribution, high drug encapsulation, and increased storage stability. It enhanced cell uptake, in vivo corneal permeation, and improved anti-inflammatory activity.	[41]
PVCL-PVA-PEG micelles	Myricetin	Dry eye and chronic anterior uveitis	Topical	PVCL-PVA-PEG micelle formulation showed high encapsulation of myricetin, no significant cytotoxicity, and good in vivo ocular tolerance. They significantly enhanced the aqueous solubility and stability of the compound and improved in vitro antioxidant and in vivo anti-inflammatory activity.	[42]
Lecithin/chitosan nanoparticles	Resveratrol	Age-related macular degeneration, diabetic retinopathy, glaucoma, cataracts.	Topical	RMLCN optimized formulation was retained on the eye surface, ensuring a sustained drug delivery.	[30]
Micelles	Resveratrol	-	Topical	Res was highly loaded into micelles. They provided more chemical stability in an aqueous solution, good short-term storage and exhibited good tolerance. Cornea permeation was also greatly improved.	[44]

Abbreviations: PVCL-PVA-PEG—polyvinyl caprolactam–polyvinyl acetate–polyethylene glycol; RMLCN—resveratrol-loaded mucoadhesive lecithin/chitosan nanoparticles; Res—resveratrol.

## Data Availability

Data sharing is not applicable.
